# The Role of Perception of Support in the Classroom on the Students’ Motivation and Emotions: The Impact on Metacognition Strategies and Academic Performance in Math and English Classes

**DOI:** 10.3389/fpsyg.2019.02794

**Published:** 2020-01-09

**Authors:** Ruben Trigueros, José M. Aguilar-Parra, Remedios Lopez-Liria, Adolfo J. Cangas, Jerónimo J. González, Joaquín F. Álvarez

**Affiliations:** ^1^Department of Psychology, University of Almería, Almería, Spain; ^2^Department of Nursing Science, Physiotherapy and Medicine, University of Almería, Almería, Spain; ^3^Department of Psychology, University of Burgos, Burgos, Spain

**Keywords:** motivation, mathematics, English, emotions, academic performance, teacher

## Abstract

According to various studies and the Cambridge University Student Barometer, Spanish students have the worst academic results in mathematics and English among the European Union countries. The objective of this study has been to analyze the influence of the teacher on motivation, positive emotions, metacognition strategies, and the academic performance of the students in those classes. We analyzed responses from 604 students of English and 547 students of mathematics, aged between 13 and 19 years. The teacher plays a relevant role in the motivation and emotions generated in the students, issues that determine decision making in the students’ learning and academic performance.

## Introduction

School failure in the secondary education stage is mainly due to the low motivation of students due to the lack of response of the educational establishment to the interests of the students (Cerda et al., 2016; Sánchez, 2016). In this sense, 22.2% of students who study mathematics during the secondary stage would not reach level 2, in the use of algorithms, formulas, procedures, or basic conventions (Goikoetxea and Jáuregui, 2008). On the other hand, according to the 2017 Cambridge University Student Barometer, the English level for Spanish adolescents is well below countries such as Denmark, Germany, France, or Italy, occupying 21st place (out of 27) in Europe (Education First, 2018). These educational levels have highlighted the marked deficiencies present in the Spanish educational system, which is sometimes unable to motivate and enthuse students toward their own learning. In this sense, teachers play a key role in promoting the students’ interest in learning and achieving academic goals (Oriol-Granado et al., 2017). For this reason, it is necessary to analyze the emotional and motivational processes inherent to the students toward math and English classes as well as the influence on learning strategies and academic achievement.

Self-determination theory (SDT) suggests the influence that the teacher can have upon their students takes place through two interpersonal styles – support for autonomy versus controlling behavior (Haerens et al., 2015). Support for autonomy refers to the encouragement by the teacher to the students’ own initiative as well as their mental and physical self-development (Hagger et al., 2007). Conversely, the use of controlling behaviors where external pressures prevail and the use of coercive means and impositions by the teacher are perceived by students as the origin of their behavior, undermining their own initiative, effort, and personal self-knowledge (Trigueros-Ramos et al., 2017). In particular, it is believed that the role the teacher adopts could influence the development of the students’ psychological needs (PNs) in a significant way.

According to SDT, these PNs constitute a series of psychological mechanisms that act as behavioral regulators for competence, autonomy, and relatedness (Deci and Ryan, 2014). Competence is the ability to skillfully perform the actions carried out; autonomy is those actions that are carried out for internal reasons, without external pressures, whereas relatedness is the feeling of belonging to a social group (Deci and Ryan, 2014). These three PNs are clearly linked to one another, so if one increases, the other will do so also; that is, students who feel more autonomous when participating in the decision-making process feel effective when performing actions and feel integrated into their social reference group, thus experiencing greater PN satisfaction. They also tend to experience self-determined motivation that is related to assimilating new information, commitment to learning, and the development of new strategies (Clayton et al., 2010). On the other hand, if students experience a feeling of abandonment during classes, minimal success in their actions, a lack of decision making, and activities that are overly monotonous or repetitive, they feel their PNs are thwarted, non-self-determined motivation that is related to activity abandonment, a lack of commitment, and the manifestation of maladaptive behaviors (Deci and Ryan, 2016).

In this way, the PN and the role played by teachers during their classes can influence student motivation (Trigueros et al., 2019c). According to SDT, there are three different types of motivation: intrinsic, extrinsic, and amotivation, ordered from more to less self-determined. Intrinsic motivation is related to behavior based on one’s own choice, the capacity for personal decision making and initiative – students tend to stick to particular actions out of the simple pleasure and enjoyment that the action provokes, facilitating behavioral adaptation that leads to self-regulation (Ricard and Pelletier, 2016). Conversely, extrinsic motivation is related to participation in events due to external pressures or acquired obligations (Ryan and Deci, 2017). Finally, amotivation means the complete absence of motivation (Ryan and Deci, 2017). These last two types of motivation lead to a lack of self-regulation concerning adaptive behavior since they tend to move away from the actions for the absence of rewards or external social recognition (Deci and Ryan, 2016). Therefore, the social environment would be fundamental for the student to experience PN satisfaction and hence a self-determined motivation (Deci and Ryan, 2016).

Despite the importance of the teacher and PNs on student motivation, emotions are also relevant to the study time and academic effort (Van den Broeck et al., 2008). Emotions constitute an evaluative assessment of an external situation, which produces both a psychological and physiological activation in the body and determine our actions, indirectly influencing academic performance (Schukajlow et al., 2017), a relationship mediated by the motivational processes (Elliot and Pekrun, 2007). In this way, positive emotions would influence the intrinsic motivation, for example, when a certain task is completed successfully or when positive expectations increase motivation to favor implementation and further performance.

The studies so far existing (Trigueros and Navarro, 2019) have made special reference to the adoption of positive adaptive behaviors generated by classes in relation to students, focusing especially on the main objective set by Organic Law 8/2013 (LOMCE), which is the adoption of adaptative habits outside school. In this sense, the LOMCE promotes interdisciplinary learning of the different subjects belonging to the curriculum. So the adoption of certain learning strategies of students can be used in one or several subjects in order that they can achieve the educational objective.

Metacognition strategies are those that allow students to observe their own learning process using various resources that serve to plan, monitor, and evaluate their own progress (Rosen et al., 2011). An example of these metacognitive strategies is the realization of concept maps, summaries, reflective reading, and others. This implies the implementation of a series of skills based on the capacities to argue, recognize different relationships, evaluate evidence and authority, issue conclusions, and make correct inferences (Aydìn, 2015). It is therefore necessary to foster educational models that try to promote the use of these strategies, since they tend to succeed in achieving academic goals by linking learning to everyday situations and promoting awareness of limitations when reasoning, thinking, and facing problems (Saiz and Fernández, 2012). The Metacognitive and Affective of Self-Regulation Learning Model (MASRL; Efklides, 2011) tries to explain the relationship between the self-regulation of cognition and motivation and its effect on behavior through the generation of strategies that regulate it. Specifically, students’ cognitive, metacognitive, and motivational characteristics lead to decisions related to commitment to a particular task and self-regulation. However, any decision can be modified or substituted depending on the task processing management and associated experiences (Reeve et al., 2007; Reeve, 2012). In such a way, students may have the belief that they are going to have to take a very easy exam, but while studying the subject, they may have the feeling that the exam may be more difficult than they originally believed so they decide to modify the way they study in order to face the exam with guarantee.

Studies focusing on students’ motivational and emotional processes toward math and English classes are scarce although there are some that have focused on motivation (Kim et al., 2014; Pham and White, 2018), self-efficiency (Skaalvik et al., 2015), or attitudes (Oroujlou and Vahedi, 2011). The Kiemer et al. (2015) study demonstrated that the importance of productive discourse in the classroom on the part of the teacher and the promotion of positive experiences regarding autonomy, competence, and relatedness favor meaningful learning through the students’ intrinsic motivation and the increase in interest toward math and science subjects. On the other hand, Kim et al. (2014) analyzed how the perception of self-efficiency and effort regulation positively mediated the effects of motivation on academic performance with high school math students who were studying in a virtual way. In another study, Soenens et al. (2012) showed that high levels of autonomy support perceived by high school students were related to a series of positive consequences such as motivation to study, learning, and academic performance. Conversely, the control strategies perceived by students were related to less motivation and vague academic expectations.

In terms of emotions, Pekrun et al. (2012), in a study on high school students, showed how positive emotions had a positive influence on motivation, the use of strategies for meaningful learning, and academic performance. On the other hand, Pekrun et al. (2017) highlighted the importance of positive emotions on the students’ academic achievement in math and vice versa. A study conducted by Pekrun et al. (2009) with 216 high school students demonstrated that intrinsic motivation predicted positive emotions, and these, in turn, predicted academic achievement and performance.

**FIGURE 1:**
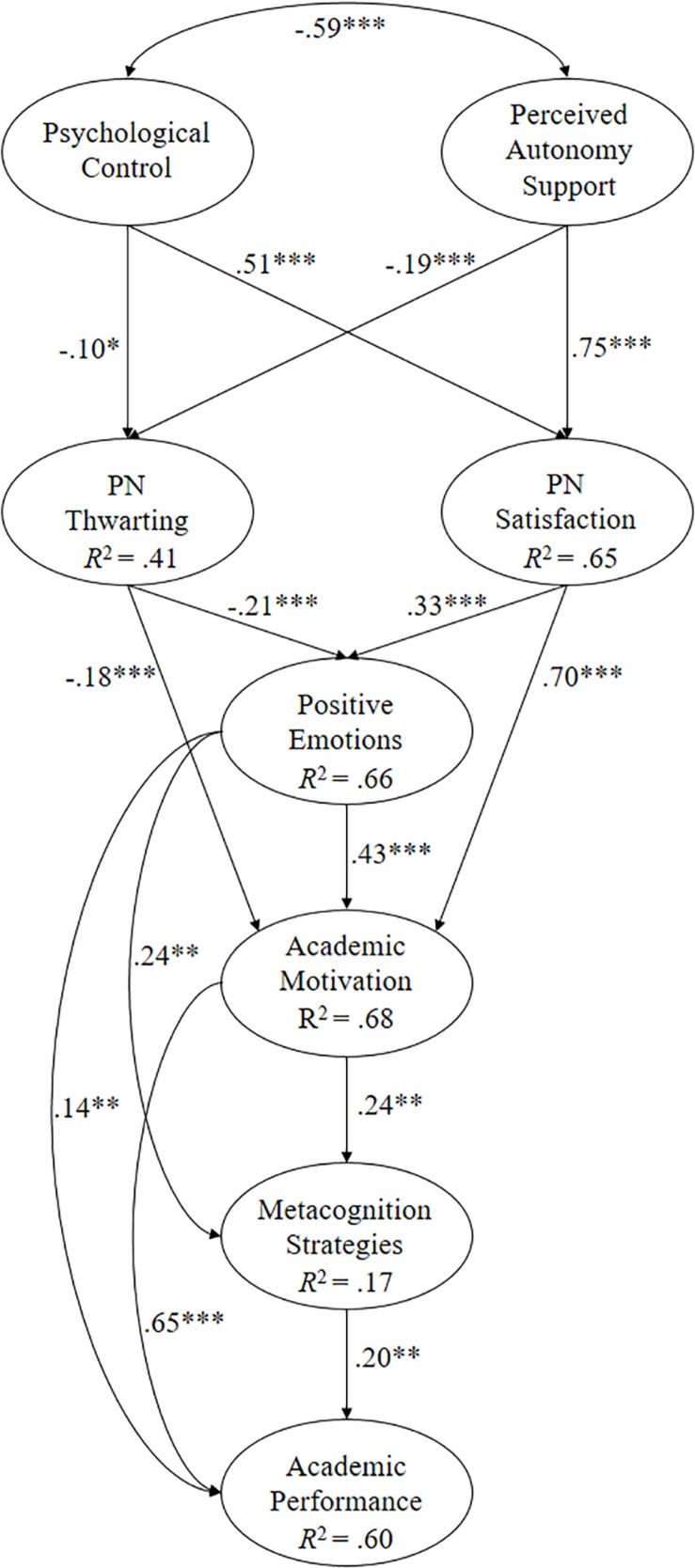
Of structural equations showing the relationships between the different variables. All parameters are standardized and statistically significant. ^∗∗∗^*p* < 0.001, ^∗∗^*p* < 0.01, and ^∗^*p* < 0.05.

**FIGURE 2:**
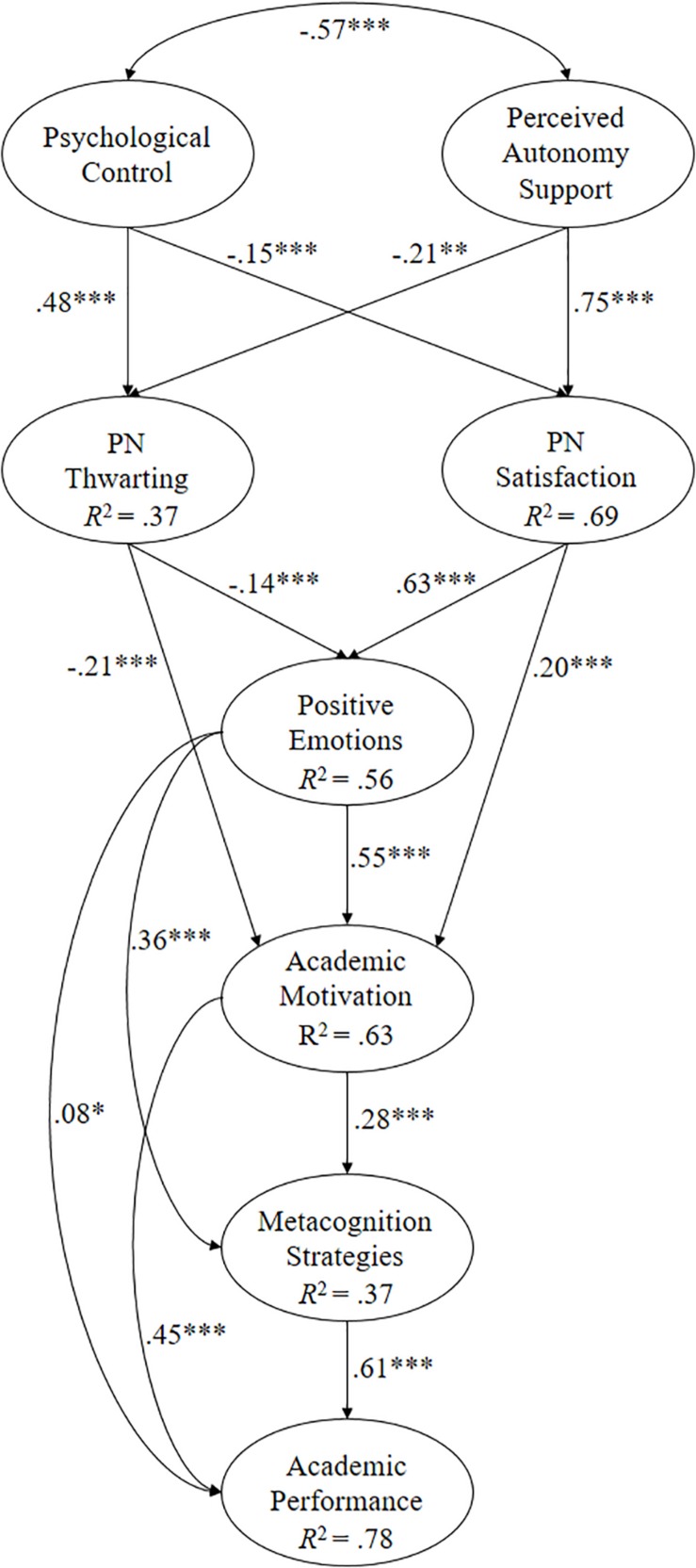
Of structural equations showing the relationships between the different variables. All parameters are standardized and statistically significant. ^∗∗∗^*p* < 0.001, ^∗∗^*p* < 0.01, and ^∗^*p* < 0.05.

The objective of this research has been to analyze how the teacher’s role influences the students’ emotional and motivational processes and their consequences regarding the use of metacognition strategies and academic performance. Two studies have been designed with independent samples, covering the entire secondary school, one for English language learners and the other for math students, posing the following hypotheses (see Figures 1, 2):

(1) The support perceived by students regarding their autonomy would positively predict PN satisfaction and negatively predict PN thwarting.
(2) Conversely, the psychological control perceived would act inversely on satisfaction and thwarting.
(3) PN satisfaction would positively predict academic motivation and positive emotions.
(4) PN thwarting would negatively predict both academic motivation and positive emotions.
(5) Positive emotions would positively predict academic motivation, metacognition strategies, and academic performance.
(6) Academic motivation would positively predict metacognition strategies and academic performance.
(7) Metacognition strategies would positively predict academic performance.

## Materials and Methods

### Participants

For the English study, 604 students participated, 321 boys and 283 girls, aged between 13 and 19 years (*M* = 15.73; *SD* = 1.30). On the other hand, the math study involved 547 students (289 boys and 258 girls) aged between 13 and 19 years (*M* = 15.94; *SD* = 1.31). With the participation of this sample, the entire secondary education is encompassed.

The groups were from different compulsory secondary education centers in Almeria Province. The inclusion criteria for the studies were voluntary participation and written informed consent given by parents (given the participants were under age).

### Instruments

#### Perceived Autonomy Support

A short version of the Teacher as Social Context Questionnaire (TASC; Belmont et al., 1988) was used. A scale consisting of eight items that assessed a single factor in perceived autonomy support (e.g., my teacher listens to my ideas) was scored using a Likert-type scale ranging from 1 (*completely disagree*) to 7 (*completely agree*).

#### Psychological Control

The psychologically controlling teaching scale (PCTS; Soenens et al., 2012) was used, in a version validated and adapted by Trigueros et al. (2020) for the physical education (PE) context. On this scale, only the word *physical education* was replaced with either English or math (in each case), with the heading “My teacher of English or mathematics.” (depending on the study). This scale consisted of seven items with a single factor, responded to with a Likert-type scale ranging from 1 (*totally disagree*) to 5 (*totally agree*).

#### Basic Psychological Needs Satisfaction

The version used by Menéndez and Fernández-Rio (2018) was validated and adapted to the Spanish PE context from Basic PNs in PE (BPN-PE; Vlachopoulos et al., 2011). The scale comprised a total of 12 items, four items corresponding to autonomy, four items corresponding to competition, and four items corresponding to relatedness. In the scale, only the word *physical education* was replaced by either English or math (in each case). The scale was preceded with the heading “In my English or math classes.” (depending on the student sample). The responses were collected on a Likert-type scale ranging from 1 (*totally disagree*) to 7 (*totally agree*).

#### Basic Psychological Needs Thwarting

This is part of the version adapted to the Spanish PE (Trigueros et al., 2019d) context from the Scale of PNs Frustration in physical exercise (EFNP; Sicilia et al., 2013). The scale was composed of a total of 12 items, divided equally into three factors (autonomy, competence, and relatedness). In the scale, only the word *physical education* was replaced by English or math (in each case). The scale was preceded with the heading “In my English or math classes” (depending on the study). The responses were collected on a Likert-type scale ranging from 1 (*totally disagree*) to 7 (*totally agree*).

#### Positive Emotions

The Emotions State in PE Classes questionnaire was used (Trigueros et al., 2019a). The questionnaire’s header was adapted to the context of the two samples. The scale consisted of a total of 32 items distributed among the eight factors – four being negative (e.g., anxiety, embarrassment, boredom, and hopelessness) and four positive (e.g., fun, pride, tranquility, and confidence), making use of the items referencing these last factors. Students had to respond to questions on a Likert-type scale ranging from 1 (*totally disagree*) to 7 (*totally agree*).

#### Academic Motivation Toward Learning

The version used was validated and adapted from the Academic Self-Regulation Scale (Vansteenkiste et al., 2009) to the Spanish context by Trigueros et al. (2019b). It consisted of 24 items, grouped into six factors that measured intrinsic motivation, integrated regulation, identified regulation, introjected regulation, external regulation, and amotivation. The students responded using a Likert-type scale ranging from 1 (*not true at all*) to 7 (*totally true*).

The relative autonomy index (RAI) was used to evaluate academic motivation. RAI was calculated as recommended by Vallerand (2007), assigning a weight to each type of motivation according to its place in the motivational continuum. The following formula was used to calculate the RAI: (3 × Intrinsic Mot.) + (2 × Integrated Reg.) + (1 × Identified Reg.) − (1 × Introjected Reg.) − (2 × External Reg.) − (3 × Amotivation). This index has proven itself to be valid and reliable in several works, being used to obtain a value for quantifying the level of self-determination.

#### Learning Focus

In order to measure the metacognition strategy, the Motivated Strategies for Learning Questionnaire (MSLQ; Pintrich et al., 1991) was used after being validated and adapted to the Spanish context by Roces et al. (1995). Only 12 items were used that made reference to metacognition strategies. The students responded using a Likert-type scale ranging from 1 (*not true at all*) to 5 (*completely true*).

#### Academic Performance

To measure academic performance, the grades obtained over the academic year were taken into account in the subjects of English and math, respectively. The grades were distributed in the following way: 1 (fail), 2 (pass), 3 (good), 4 (very good), and 5 (excellent).

### Procedure

This study followed all procedural ethics with regard to the ethical treatment of human participants. It has requested permission to conduct this study, which was approved by the Bioethics Committee in Human Research of the University of Almería (Ref: UALBIO 2019/014). Furthermore, we have obtained written informed consent from the parents/legal guardians of all participants of the study, because they were underage students. Also, previously, the parents/legal guardians were informed of the objective and the procedure of this study in writing.

First, we contacted various educational centers in Almeria Province, asking for permission to give the questionnaires to their students after informing them of the study objectives. Then the scales were administered to the participants under the supervision of a survey expert (a member of the research group), who explained and resolved any queries that arose when filling out the questionnaires. The time estimated to complete the questionnaires was around 25 min.

### Data Analysis

First, the descriptive statistics were calculated, and with the Pearson correlation, the correlation between the study variables was analyzed. Subsequently, the hypothesized predictive model was tested using a structural equation model (SEM). To test the effects of mediation between the model variables, the premises established by Baron and Kenny (1986) were taken into account: (a) significant correlations between the independent and dependent variables; (b) significant correlations between the independent variable and the mediators; (c) significant correlations between the mediators and the dependent variable; (d) the prior significant relationship between the independent and dependent variables that ceases being significant when relationships between the independent variable and the mediators and between the mediators and the dependent variable are controlled.

For the SEM, the maximum likelihood estimation method was used with the bootstrapping procedure in the AMOS 19 statistical package. The following indexes were used to analyze the model’s goodness of fit: the chi square coefficient, the chi square to its degrees of freedom (χ^2^/*df*), the comparative fit index (CFI), the incremental fit index (IFI), the root mean square error of approximation (RMSEA) plus its 90% confidence interval (CI), and the standardized root mean square residual (SRMR). Generally, values are considered acceptable if they are below 5 χ^2^/*df* (Bentler, 1990), likewise for CFI and IFI values equal to or above 0.90, RMSEA values below 0.08, and SRMR values of 0.06 or less (Hu and Bentler, 1999).

## Results

### Preliminary Analysis

In Table 1, one can observe that the average scores for the English students were moderate. Only psychological control and PN frustration were below the questionnaire’s arithmetic mean. The same thing occurred for the math students (see Table 2).

**TABLE 1:** Descriptive statistics and the correlation between the English model variables.

**Variables**	***M***	***DT***	**1**	**2**	**3**	**4**	**5**	**6**	**7**	**8**
1. Perceived autonomy support	4.48	1.34		–0.57^∗∗^	0.64^∗∗^	–0.40^∗∗^	–0.59^∗∗^	0.59^∗∗^	0.27^∗∗^	0.49^∗∗^
2. Psychological control	1.82	1.06			–0.43^∗∗^	0.54^∗∗^	–0.51^∗∗^	–0.54^∗∗^	–0.27^∗∗^	–0.44^∗∗^
3. PNS	4.08	1.21				–0.45^∗∗^	0.65^∗∗^	0.66^∗∗^	0.28^∗∗^	0.55^∗∗^
4. PNT	2.59	1.21					–0.48^∗∗^	–0.54^∗∗^	–0.18^∗∗^	–0.44^∗∗^
5. Positive emotions	4.95	1.51						0.79^∗∗^	0.35^∗∗^	0.67^∗∗^
6. Academic motivation	11.49	16.05							0.35^∗∗^	0.77^∗∗^
7. Metacognition strategy	3.26	0.72								0.31^∗∗^
8. Academic performance	3.78	1.31								

*^∗∗^*p* < 0.01. PNT, psychological needs thwarting; PNS, psychological needs satisfaction.*

**TABLE 2:** Descriptive statistics and the correlation between the math model variables.

**Variables**	***M***	***DT***	**1**	**2**	**3**	**4**	**5**	**6**	**7**	**8**
1. Perceived autonomy support	4.44	1.29		–0.52^∗∗^	0.63^∗∗^	–0.42^∗∗^	0.57^∗^	0.55^∗∗^	0.36^∗∗^	0.46^∗∗^
2. Psychological control	1.81	1.02			−0.43^∗^	0.55^∗∗^	–0.50^∗∗^	–0.49^∗∗^	–0.27^∗∗^	–0.40^∗∗^
3. PNS	4.33	1.16				–0.58^∗∗^	0.68^∗∗^	0.69^∗∗^	0.45^∗^	0.57^∗∗^
4. PNT	2.64	1.34					–0.52^∗∗^	–0.52^∗∗^	–0.37^∗∗^	–0.55^∗∗^
5. Positive emotions	4.93	1.46						0.78^∗∗^	0.53^∗∗^	0.63^∗∗^
6. Academic motivation	11.58	15.43							0.55^∗∗^	0.73^∗∗^
7. Metacognition strategy	4.02	1.11								0.80^∗∗^
8. Academic performance	3.82	1.29								

*^∗^*p* < 0.05; ^∗∗^*p* < 0.01. PNT, psychological needs thwarting; PNS, psychological needs satisfaction.*

The reliability analysis using Cronbach’s alpha for the English students produced a value of 0.82 for perceived psychological control, 0.85 for perceived autonomy support, 0.89 for PN thwarting, 0.92 for PN satisfaction, 0.94 for positive emotions, and 0.82 for the metacognition strategy.

The reliability analysis using Cronbach’s alpha for math students produced a value of 0.96 for perceived psychological control, 0.92 for perceived autonomy support, 0.85 for PN thwarting, 0.72 for PN satisfaction, 0.95 for the metacognition strategy, and 0.92 for positive emotions.

After the Pearson correlation analysis was conducted in both studies [math students (Table 2) and English students (Table 1)], it was observed how psychological control related positively with PN thwarting and negatively for perceived autonomy support, PN satisfaction, positive emotions, academic motivation, metacognition strategy, and academic performance.

Perceived autonomy support correlated negatively with PN thwarting and positively with PN satisfaction, positive emotions, academic motivation, metacognition strategy, and academic performance.

PN thwarting correlated negatively with respect to PN satisfaction, positive emotions, academic motivation, metacognition strategy, and academic performance.

Conversely, PN satisfaction correlated positively with positive emotions, academic motivation, metacognition strategy, and academic performance.

Positive emotions correlated positively with the RAI academic, metacognition strategy, and academic performance.

Academic motivation correlated positively with respect to the metacognition strategy and academic performance; and finally, the metacognition strategy correlated positively with academic performance.

### Structural Equation Model

When the predictive relationship model hypothesized for English students was tested (Figure 1), the following fit indices were revealed: χ^2^(122, *N* = 604) = 512.27, *p* < 0.001; χ^2^/*df* = 4.20; CFI = 0.95; IFI = 0.95; RMSEA = 0.073 (90% CI = 0.066–0.079); and SRMR = 0.043.

The following are the relationships obtained between the different factors making up the English model (Figure 1):

(a) The correlation between psychological control and support for autonomy was negative (β = −0.59, *p* < 0.001).
(b) Psychological control positively predicted PN thwarting (β = 0.51, *p* < 0.001) and, in turn, negatively predicted PN satisfaction (β = −0.10, *p* < 0.05).
(c) Autonomy support positively predicted PN satisfaction (β = 0.75, *p* < 0.001) and negatively predicted PN thwarting (β = −0.19, *p* < 0.001).
(d) PN satisfaction positively predicted positive emotions (β = 0.70, *p* < 0.001) and academic motivation (β = 0.33, *p* < 0.001).
(e) PN thwarting negatively predicted positive emotions (β = −0.21, *p* < 0.001) and academic motivation (β = −0.18, *p* < 0.001).
(f) Positive emotions positively predicted academic motivation (β = 0.43, *p* < 0.001), the metacognition strategy (β = 0.24, *p* < 0.01), and academic performance (β = 0.14, *p* < 0.01).
(g) Academic motivation positively predicted the metacognition strategy (β = 0.20, *p* < 0.01) and academic performance (β = 0.65, *p* < 0.001).
(h) The metacognition strategy positively predicted academic achievement (β = 0.30, *p* < 0.05).

When the predictive relationship model hypothesized for the math students was tested (Figure 2), the following fit indices were revealed: χ^2^(122, *N* = 547) = 383.40, *p* < 0.001; χ^2^/*gl* = 3.14; CFI = 0.97; IFI = 0.97; RMSEA = 0.063 (90% CI = 0.056–0.070); SRMR = 0.047.

In contrast, the relationships obtained between the different factors that make up the math model are described (Figure 2):

(a) The correlation between psychological control and support for autonomy was negative (β = −0.57, *p* < 0.001).
(b) Psychological control positively predicted PN thwarting (β = 0.48, *p* < 0.001) and, in turn, negatively predicted PN satisfaction (β = 0.15, *p* < 0.001).
(c) Support for autonomy positively predicted PN satisfaction (β = 0.75, *p* < 0.001) and, in turn, negatively predicted PN thwarting (β = −0.21, *p* < 0.01).
(d) PN satisfaction positively predicted positive emotions (β = 0.63, *p* < 0.001) and academic motivation (β = 0.20, *p* < 0.001).
(e) PN thwarting negatively predicted positive emotions (β = −0.21, *p* < 0.001) and academic motivation (β = −0.14, *p* < 0.001).
(f) Positive emotions positively predicted academic motivation (β = 0.55, *p* < 0.001), the metacognition strategy (β = 0.36, *p* < 0.001), and academic performance (β = 0.08, *p* < 0.05).
(g) Academic motivation positively predicted the metacognition strategy (β = 0.28, *p* < 0.001) and academic performance (β = 0.45, *p* < 0.001).
(h) The metacognition strategy positively predicted academic achievement (β = 0.61, *p* < 0.001).

## Discussion

### General Discussion

The present study analyzes the areas of math and English – how the students’ perception of the interpersonal teaching style affects the PNs, positive emotions, academic motivation, use of metacognition strategies, and academic performance, that is to say, how the teacher’s role can influence the students in terms of offering them autonomy support rather than psychological control, with regard to PN frustration or satisfaction as well as its effects on academic motivation and positive emotions. The study considered both the positive and negative aspects that may be present in math and English classes – issues such as the teacher’s controlling style or the PN satisfaction and frustration, which are frequently experienced by the students.

The results of this study show that support for autonomy positively predicts PN satisfaction and negatively predicts PN thwarting; in contrast, perceived control acts inversely on PN thwarting and satisfaction. However, studies dealing with these variables in the areas of math and English have not been found, only in PE. Accordingly, a study by Haerens et al. (2015) showed how the perception of supported autonomy and teaching control, as well as PN satisfaction and frustration, comprise different constructs that are disparately related to student motivation; that is to say, perceived autonomy support was related to self-determined motivation and PN satisfaction, which acted as a mediator for this association while the perception of controlled teaching was mainly related to non-self-determined motivation and amotivation, with PN thwarting acting as a mediator in this relationship. On the other hand, the study by Hein et al. (2015) showed how the students’ perception of the controlling teaching style had a positive influence on feelings of anger and harassment, which promoted PN thwarting. In contrast, Yu et al. (2015) showed how support for autonomy had a positive influence on PN satisfaction, giving rise to increased student commitment. These studies show the importance of teaching and its influence on the motivational, social, and psychological development of the students as well as their PNs, since they are psychological mechanisms that act as behavioral regulators (Hagger et al., 2003; Haerens et al., 2015; Hein et al., 2015; Yu et al., 2015).

The results of this research have also shown that PN frustration negatively predicts positive emotions and academic motivation, whereas PN satisfaction inversely predicts them. Accordingly, a study by Cantú-Berrueto et al. (2016) showed how PN satisfaction led to students experiencing self-determined motivation toward PE classes while PN frustration predicted non-self-determined motivation. Similarly, Trigueros et al. (2019c) showed how PN satisfaction positively predicted motivation and negatively predicted shame; however, frustration of these needs acted inversely on the relationships. According to all these studies, PN satisfaction and/or thwarting influence emotions (Wei et al., 2005; Taylor et al., 2010; Tessier et al., 2010; Bartholomew et al., 2011), information that Wei et al. (2005) corroborate, showing how PN satisfaction negatively influences depression, stress, and shame. Likewise, Tessier et al. (2010) showed how PN satisfaction was associated positively with certain emotions such as fun, joy, and enthusiasm but negatively with boredom and disinterest. The research results described are in accordance with the principles set forth in the SDT regarding the relationship between PN and positive emotions (Deci and Ryan, 2011); this is due to the effects of PN on emotional and psychological well-being (Tessier et al., 2010; Trigueros et al., 2019c). This study is based on the idea put forward by Deci and Ryan (2014) that PN thwarting can trigger a series of maladaptive consequences that lead to disinhibition and/or behavior that is contrary to personal well-being, the inverse occurring in the case of PN satisfaction.

On the other hand, the analyzed results indicate that positive emotions predict academic motivation, meaningful learning strategies, and academic achievement in a positive way, information that is in line with other studies carried out in the university student population, where positive emotions showed this relationship with intrinsic motivation, self-regulation of academic behavior, and academic performance (Villavicencio and Bernardo, 2013; Pekrun et al., 2017). These findings indicate that student emotions are related to their control, motivation, use of learning strategies, and academic performance (Pekrun et al., 2017).

Finally, motivation showed a positive relationship with metacognition strategies and academic performance. Similarly, metacognition strategies showed a positive relationship with academic achievement. Such findings have been described in research on the university student population (Wolters and Hussain, 2015) and students engaged in distance learning (Broadbent, 2017). These could be explained by the fact that metacognitive strategies are procedures that facilitate information processing by selecting, organizing, and regulating cognitive processes (Karpicke et al., 2009). For their use, it is necessary that students show great interest in the subject, namely, an intrinsic motivation toward it, since it requires conscious planning and use of these strategies to facilitate academic performance (Zimmerman, 2008).

Among the limitations, it is necessary to point out that this is a correlational study, so it does not allow one to extrapolate the cause–effect relationships – the results obtained could have different interpretations depending on the context. Furthermore, in the present study, we have not been able to carry out a comparative study of the results according to the school year or the age of the students. In addition, the SEMs have the limitation that their relationships are unidirectional. On the other hand, there may be other factors that significantly influence student achievement and academic performance, in addition to the variables considered. Finally, in the future, comparative studies should be carried out between the different countries of the European Union in relation to the motivation, emotion, and metacognitive strategies of high school students in the areas of math and English, being able to introduce new variables such as emotional intelligence and critical thinking, because those factors infer on the performance and motivation according to different studies (Homayouni, 2011; Galla and Wood, 2012).

### Discussion of Math Context

The present study is pioneering in jointly demonstrating the importance of the variables of emotions, motivation for learning, and the students’ attributional style in the area of math. In this sense, the practices derived from the study point to the need to incorporate motivational and emotional components for the training of students and teachers. It is important to provide educators with information on the role of motivation and positive emotions in the success and adaptation of students in the educational context and on how to develop the internal attributional style of their pupils. Teachers, and parents, must provide learning environments that promote autonomy over external control. When learning is achieved through procedures that support the adolescents’ involvement, the sense of self-determination and understanding of the material to be learned are enhanced. Teachers should accompany students in the learning process by transmitting their passion and enthusiasm for knowing, promoting feelings of self-efficacy and academic self-competence as the basis for educational success.

### Discussion of English Context

This study contributes to the area of teaching a foreign language in which the motivational, emotional, and attributional processes of secondary school students in that area have been taken into account. In this way, valuable pedagogical implications can be deduced so that teachers can integrate into their teaching situations that favor student reflection before, during, and after entering the classroom. It is vital, for example, that teachers take into account the following:

(1) The importance and impact of the classroom atmosphere affect students in their interest, enthusiasm, commitment, and motivation during instruction or class time. Thus, we suggest to teachers, based on the current study and what we know from previous best practice studies, the following recommendations: creating an atmosphere in class that increases comfort and confidence and developing good classroom relationships. To achieve this, the teacher should be accessible, not distant or intimidating, and should understand students’ mistakes and doubts; likewise, the teacher should try to connect and interact with students on a more personal level.
(2) The program, including both content and organization, is another important point that affects students’ levels of commitment and motivation, and therefore, teachers should consider every aspect of it in relation to the impact on instruction and what takes place in class. The organization of the program, including homework and time spent on corrections, should be considered relevant.
(3) Students’ academic performance should be assessed by teachers in a way that can better reflect what has been taught and addressed during the regular class period, rather than creating additional challenges or challenges.
(4) Teachers should use different expressions and vocabulary to explain, which allows students to activate their previous knowledge and use it to construct and understand what is new.

## Conclusion

Ultimately, this model helps us to understand the emotional and motivational processes that favor academic performance in the subjects of English and math, demonstrating good robustness in the university environment. In addition, the importance of teaching based on support for student autonomy, in order to increase their interest and motivation toward these subjects, is highlighted. In this way, students show more willingness to use different strategies for meaningful learning, resulting in increased learning, and academic performance.

## Data Availability Statement

The datasets generated in this article are not publicly available. Requests to access the datasets should be directed to corresponding author (JA-P).

## Author Contributions

All authors listed have made a substantial, direct and intellectual contribution to the work, and approved it for publication.

## Conflict of Interest

The authors declare that the research was conducted in the absence of any commercial or financial relationships that could be construed as a potential conflict of interest.
